# Trend analysis of disability adjusted life years due to cardiovascular diseases: results from the global burden of disease study 2019

**DOI:** 10.1186/s12889-021-11348-w

**Published:** 2021-06-29

**Authors:** Fatemeh Masaebi, Masoud Salehi, Maryam Kazemi, Nasim Vahabi, Mehdi Azizmohammad Looha, Farid Zayeri

**Affiliations:** 1 Department of Biostatistics, School of Allied Medical Sciences, Shahid Beheshti University of Medical Sciences, Tehran, Iran; 2 Health Management and Economics Research Center and Department of Biostatistics, School of Public Health, Iran University of Medical Sciences, Tehran, Iran; 3 Informatics Institute, University of Florida, Gainesville, FL USA; 4 Prevention of Cardiovascular Disease Research Center, Shahid Beheshti University of Medical Sciences, Tehran, Iran

**Keywords:** Cardiovascular diseases, Disability-adjusted life years, Human development index, Trend analysis

## Abstract

**Background:**

Cardiovascular diseases (CVDs) are the number one cause of global mortality representing about one third of all deaths across the world. The objective of the present study was to model the global trend in disability-adjusted life years (DALY) and its components due to CVD over the past three decades. We also aimed to evaluate the longitudinal relationship between CVD DALY and Human Development Index (HDI) in this period of time.

**Methods:**

The age-standardized rates of years lost due to disability (YLD), years of life lost (YLL) and DALY were extracted for cardiovascular diseases from the Global Burden of Disease (GBD) Study 2019 in years 1990 to 2019. Additionally, the United Nations Development Programme (UNDP) database was used to retrieve HDI values for all world countries at the same period time. The trend analysis was performed using the joinpoint regression model.

**Results:**

The obtained revealed a significant downward trend for DALY and its components with the average annual percent change of − 1.0, − 0.3 and − 1.1 per 100,000 population, respectively for DALY, YLD and YLL. We also found that countries with high/very high levels of HDI have remarkably experienced steeper declining slope of trend than those in lower levels of HDI over the study period.

**Conclusions:**

Although the observed decreasing trend of CVD burden is a hopeful message for all world countries, the considerable gap in slope of trend between richer and poorer parts of the world is a serious alarm for health policy makers. Regarding this, there is an urgent need to put more efforts on implementing preventive programs, improving the level of patients’ care and providing efficient treatment, especially in regions with lower levels of HDI.

## Introduction

Cardiovascular diseases (CVDs) are the leading cause of mortality and one of the most important causes of disability in the entire world [1]. CVD is a general term for a class of diseases that affect the heart or blood vessels. Coronary artery disease, heart attack, stroke, arrhythmia, heart failure, heart valve problems, congenital heart defects, cardio myopathy and peripheral artery disease are some the most common types of cardiovascular disease [2]. Despite marked improvements in prevention, early diagnosis and treatment, CVDs still impose huge socio-economic burden on health systems and community. In the last three decades, the global prevalence of CVDs had an increase of 93% (from 271 million in 1990 to 523 million in 2019) and the CVD mortality has risen about 54% (from 12.1 million in 1990 to 18.6 million in 2019) which represents about one-third of the annual deaths across the world [3]. While non-communicable diseases (NCDs) are responsible for 60% of disability-adjusted life years (DALY) globally, about one-fourth of this burden is attributable to cardiovascular diseases [4].

Regarding the 25 × 25 Global Action Plan suggested by the World Health Organization (WHO) in 2013 which targets to reduce premature deaths from NCDs by 25% by 2025, the world countries have focused on preventing and managing CVD (as one of the NCDs with the highest global burden) and its related risk indicators (tobacco use, diet, physical activity, and excessive alcohol consumptions) [5]. Although high-income countries could meet these targets by 2025, limited access to health care and treatment, insufficient health funding, poor governance and more focus on curative services rather than preventive actions make these goals hard to achieve in low and middle-income countries [4]. In this context, investigating the long-term patterns of CVD burden components seems to be highly crucial, especially in terms of socio-economic level of world countries. The obtained results might help the health policy makers to know in which levels of socio-economic status the CVD burden is increasing and in which levels it has stable or decreasing pattern.

Reviewing the revealed works in this field shows that there are few published articles about the long-term trend of CVD burden and its association with socio-economic status of the world countries. For instance, a study in 2015 showed that in countries with higher levels of socio-demographic index (SDI), CVD mortality decreases sharply for both the male and female populations [6]. In another study in 2020, the researchers concluded that the CVD burden is rising not only in nearly all low and middle-income countries but also in some parts of high-income regions [3]. In addition, reviewing the statistical methods of these studies shows that almost all of them have used descriptive approaches to illustrate the trend of CVD burden and its relationship with social or economic indices. Regarding this, we decided to conduct the current study to explore the global trends of years lost due to disability (YLD), years of life lost (YLL) and DALY in a long-term period (from 1990 to 2019) and investigate the longitudinal relationship between CVD burden indices and human development index (HDI) as a key indicator for evaluating the socio-economic status of the world countries. To capture the observed change points in the trend of CVD burden indices, we used the joinpoint regression model as a well-known inferential technique in analyzing the trend of longitudinal outcome variables.

## Methods

### GBD data set

The Global Burden of Disease (GBD) Study is the most comprehensive, multi-institutional and multi-individual global collaborative epidemiological research which annually estimates the burden of 369 diseases and injuries (including incidence, prevalence, mortality, YLL, YLD and DALY rates) for more than 200 world countries and territories by sex and age group from 1990 to the present. Findings from the GBD data can be used by the decision makers at the global, regional, national, and local levels to get a better understanding about health trends and improve their policy over time. In this study, we extracted the age-standardized CVD YLD, YLL and DALY rates per 100,000 people from the GBD study 2019. More detailed information about GBD study 2019 and methods of standardization can be found elsewhere [7].

### HDI data set

The Human Development Index (HDI), introduced by a Pakistani economist Mahbub ul Haq, is a summary statistic which is calculated as the geometric mean of four different indicators: life expectancy, expected years of schooling for school-age children, mean years of schooling for adults aged 25 years and more, and Gross National Income per capita. These indicators are combined in a statistics which requires transformation into an index between 0 and 1. All countries in the world can utilize this index as a helpful reference for monitoring their long-term progress in mean level of social and economic development. The United Nations Development Programme (UNDP)'s Human Development Report Office classifies the world countries according to their HDI values into four different levels: less than 0.550 for low human development, 0.550–0.699 for medium human development, 0.700–0.799 for high human development and 0.800 or higher for very high human development. In this study, we extracted the HDI data for 185 countries between 1990 and 2019 from the UNDP website [8]. In addition, to simplify the statistical analysis, we combined the countries with HDI levels of low and medium in a category and levels of high and very high in another category to form a binary explanatory variable in the modeling process.

### Statistical analysis

To describe the trend of cardiovascular disease YLD, YLL and DALY rates, the mean and its 95% confidence interval were reported globally, by gender and HDI level. In addition, to identify the pattern of changes in these three indices, joinpoint regression analysis was used. For a set of *n* observations (*t*_1_, *y*_1_), (*t*_2_, *y*_2_), …, (*t*_*n*_, *y*_*n*_), the joinpoint regression model can be written as:

$$ {y}_i={\beta}_0+{\beta}_1{t}_i+{\gamma}_1{\left({t}_i-{\tau}_1\right)}^{+}+\dots +{\gamma}_k{\left({t}_i-{\tau}_K\right)}^{+}+{\varepsilon}_i,i=1,\dots .,n $$

where *t*_*i*_ indicates the time points (1990, 1991, …, 2019) and *y*_*i*_ represents the CVD burden indices (YLD, YLL and DALY rates). Here, *τ*_*k*_, *k* = 1, 2, …, *K* represents the change point location, *K* shows the number of change points, *β*_0_, *β*_1_ and *γ*_1_, …, *γ*_*k*_ indicate the regression coefficients and *ε*_*i*_ is the model error term. The notation (*t*_*i*_ − *τ*_*k*_)^+^ = *t*_*i*_ − *τ*_*k*_ if *t*_*i*_ − *τ*_*k*_ > 0, and (*t*_*i*_ − *τ*_*k*_)^+^ = 0 otherwise. By fitting the joinpoint regression we can calculate the Annual Percent Change (APC) in rates between the estimated change points. To do this, the log transform of the model is utilized and the APC from year *τ*_*j*_ to year (*τ*_*j*_ + 1) can be calculated as:

$$ APC=100\times \left(\mathit{\exp}\left({\beta}_1+{\gamma}_1+{\gamma}_2+\dots +{\gamma}_j\right)-1\right) $$

For each fitted joinpoint regression model, the Average Annual Percent Change (AAPC) can be estimated as a weighted mean of the estimated APCs by using the segment lengths as weights [9]. The 95% confidence interval for APCs and AAPCs were also computed to evaluate the statistical significancy of the estimates. The joinpoint regression analysis was performed using the joinpoint software version 4.8.0.1 [10].

## Results

**Fig. 1:**
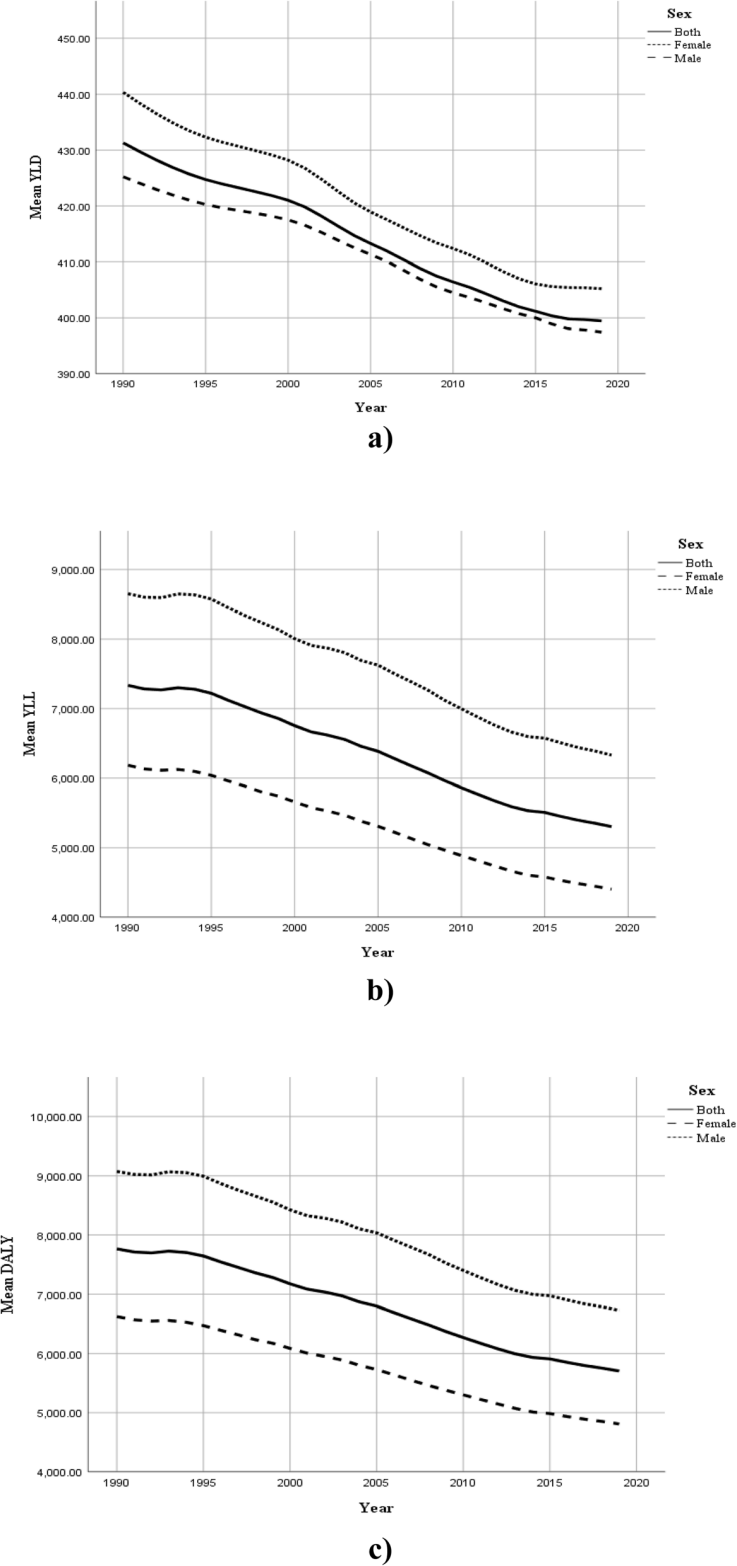
Global trend of CVD burden by gender from 1990 to 2019; **a**) YLD, **b**) YLL and **c**) DALY

In this study, the burden of cardiovascular diseases for 194 world countries was extracted from 1990 to 2019. Table 1 includes the descriptive statistics for CVD DALY and its components (YLD and YLL) by gender in 1990, 1995, 2000, 2005, 2010, 2015 and 2019. In addition, Fig. 1 displays the trend of these indices during the study period. At a glance, two main conclusions can be made about the trend of CVD burden in the entire world. First, all the three indices had a downward trend over the study period. In other words, world countries have experienced lower burden of CVD in 2019 compared to the starting point of the study. Second, female populations of the world have experienced higher rates of YLD than males, while men had higher YLL and DALY than women.

**Table 1:** Descriptive statistics for CVD DALY, YLD and YLL rates per 100,000 by gender from 1990 to 2019

Index	Gender	1990	1995	2000	2005	2010	2015	2019
**YLD**	**Male**	425.22^**a**^ (410.89, 439.54)	420.26 (406.02, 434.51)	417.49 (403.16, 431.83)	411.31 (397.05, 425.56)	404.49 (390.39, 418.58)	400.01 (386.06, 413.95)	397.44 (383.66, 411.22)
	**Female**	440.36 (424.23, 456.48)	432.32 (416.03, 448.60)	428.19 (411.75, 444.64)	418.93 (402.48, 435.37)	412.38 (395.98, 428.77)	406.07 (389.73, 422.41)	405.23 (389.06, 421.40)
	**Both**	431.30 (416.35, 446.26)	424.72 (409.71, 439.74)	421.01 (405.91, 436.11)	413.28 (398.21, 428.35)	406.43 (391.49, 421.37)	401.17 (386.30, 416.05)	399.46 (384.76, 414.16)
**YLL**	**Male**	8650.91 (8170.94, 9130.87)	8574.12 (8024.74, 9123.5)	8007.57 (7462.86, 8552.28)	7626.03 (7037.59, 8214.48)	6997.04 (6457.35, 7536.73)	6573.84 (6058.63, 7089.06)	6330.48 (5841.01, 6819.95)
	**Female**	6184.1 (5840.35, 6527.85)	6039.85 (5657.26, 6422.44)	5658.88 (5269.96, 6047.81)	5308.84 (4904.15, 5713.53)	4888.01 (4497.76, 5278.26)	4578.36 (4202.64, 4954.08)	4403.25 (4047.37, 4759.13)
	**Both**	7334.95 (6940.15, 7729.74)	7220.13 (6775.23, 7665.03)	6754.28 (6306.46, 7202.10)	6385.05 (5910.94, 6859.17)	5863.24 (5417.46, 6309.02)	5508.33 (5078.31, 5938.34)	5304.35 (4895.49, 5713.21)
**DALY**	**Male**	9076.13 (8587.72, 9564.54)	8994.38 (8436.40, 9552.36)	8425.07 (7871.51, 8978.63)	8037.34 (7440.29, 8634.39)	7401.53 (6853.08, 7949.97)	6973.85 (6449.797, 7497.9)	6727.92 (6229.58, 7226.26)
	**Female**	6624.46 (6272.56, 6976.36)	6472.16 (6080.59, 6863.74)	6087.08 (5688.46, 6485.69)	5727.764 (5313.34, 6142.18)	5300.38 (4899.93, 5700.83)	4984.43 (4598.65, 5370.21)	4808.48 (4442.45, 5174.50)
	**Both**	7766.25 (7362.92, 8169.58)	7644.85 (7190.89, 8098.82)	7175.29 (6718.03, 7632.55)	6798.34 (6314.86, 7281.81)	6269.67 (5814.29, 6725.06)	5909.50 (5469.88, 6349.12)	5703.81 (5285.30, 6122.32)

^a^mean (95% CI)

**Fig. 2:**
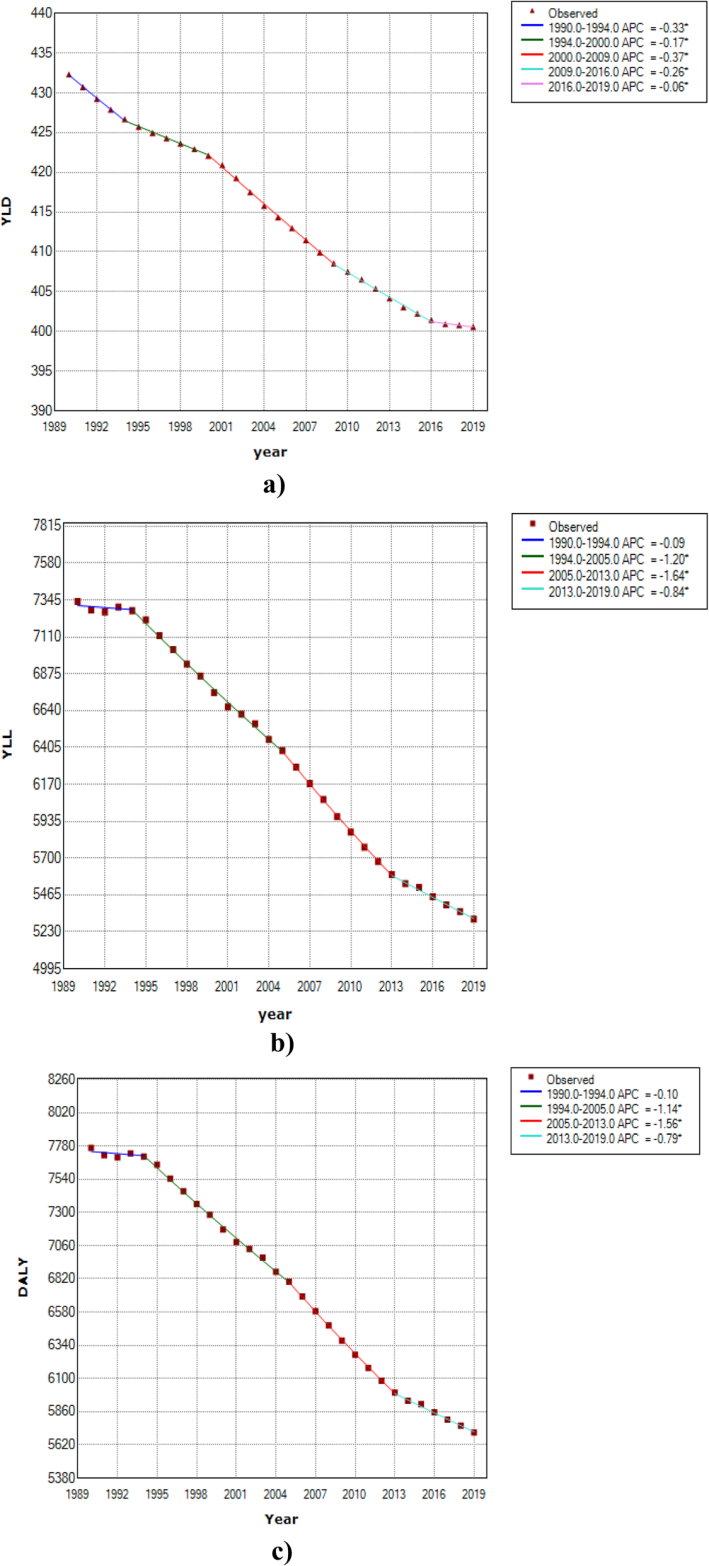
Results of joinpoint regression models for analyzing the global trend of CVD burden from 1990 to 2019; **a**) YLD, **b**) YLL and **c**) DALY

To model the behavior of CVD burden over the study period, we fitted three joinpoint regression models to YLD, YLL and DALY rates per 100,000 for total population of the world countries (regardless of gender). Table 2 and Fig. 2 show the obtained results. Regarding the estimates for YLL rates, we can observe three joinpoints in 1994, 2005 and 2013, which lead to four periods with different trends. Also, the estimated APCs show annual percent changes of − 0.1, − 1.2, − 1.6 and − 0.8 in YLL rates, respectively in the time intervals of 1990–1994, 1994–2005, 2005–2013 and 2013–2019. Of note, the only non-significant APC is related to the first interval (1990–1994) with very slight reduction in the rate of global YLL. The maximum reduction can be seen in the third period (2005–2013) with an APC of − 1.6 per 100, 000. The fitted joinpoint regression model to DALY rates shows a similar behavior with the results of the fitted model to YLL data. From the results of the fitted model to YLD rates, four joinpoints in 1994, 2000, 2009 and 2016 could be seen which lead to five periods with different trends. Also, the estimated APCs indicate significant annual percent changes of − 0.3, − 0.2, − 0.4, − 0.3 and − 0.1 in YLD rates, respectively in the time intervals of 1990–1994, 1994–2000, 2000–2009, 2009–2016 and 2016–2019. The maximum reduction is related to the third time interval (2000–2009) with an APC of − 0.4 per 100, 000. Moreover, the estimated AAPCs from these models imply a mean annual fall of 1.1, 0.3 and 1.0 per 100, 000, respectively in YLL, YLD and DALY rates for global population of the world over the study period.

**Table 2:** Results of the Joinpoint regression models for trend analysis of CVD DALY, YLD and YLL rates in total population of the world countries from 1990 to 2019

Segments	YLD		YLL		DALY	
	Time interval	APC (95% CI)	Time interval	APC (95% CI)	Time interval	APC (95% CI)
Trend 1	1990–1994	−0.3^a^ (− 0.4, − 0.3)	1990–1994	−0.1 (− 0.3, 0.1)	1990–1994	−0.1 (− 0.3, 0.1)
Trend 2	1994–2000	− 0.2^a^ (− 0.2, − 0.1)	1994–2005	− 1.2^a^ (− 1.2, − 1.1)	1994–2005	− 1.1^a^ (− 1.2, − 1.1)
Trend 3	2000–2009	− 0.4^a^ (− 0.4, − 0.4)	2005–2013	− 1.6^a^ (− 1.7, − 1.6)	2005–2013	− 1.6^a^ (− 1.6, − 1.5)
Trend 4	2009–2016	− 0.3^a^ (− 0.3, − 0.2)	2013–2019	−0.8^a^ (− 0.9, − 0.7)	2013–2019	−0.8^a^ (− 0.9, − 0.7)
Trend 5	2016–2019	−0.1^a^ (− 0.1, − 0.0)	–	–	–	–
AAPC	1990–2019	− 0.3^a^ (− 0.3, − 0.3)	1990–2019	− 1.1^a^ (− 1.1, − 1.0)	1990–2019	− 1.0^a^ (− 1.1, − 1.0)

^a^ Significant at alpha = 0.05

**Fig. 3:**
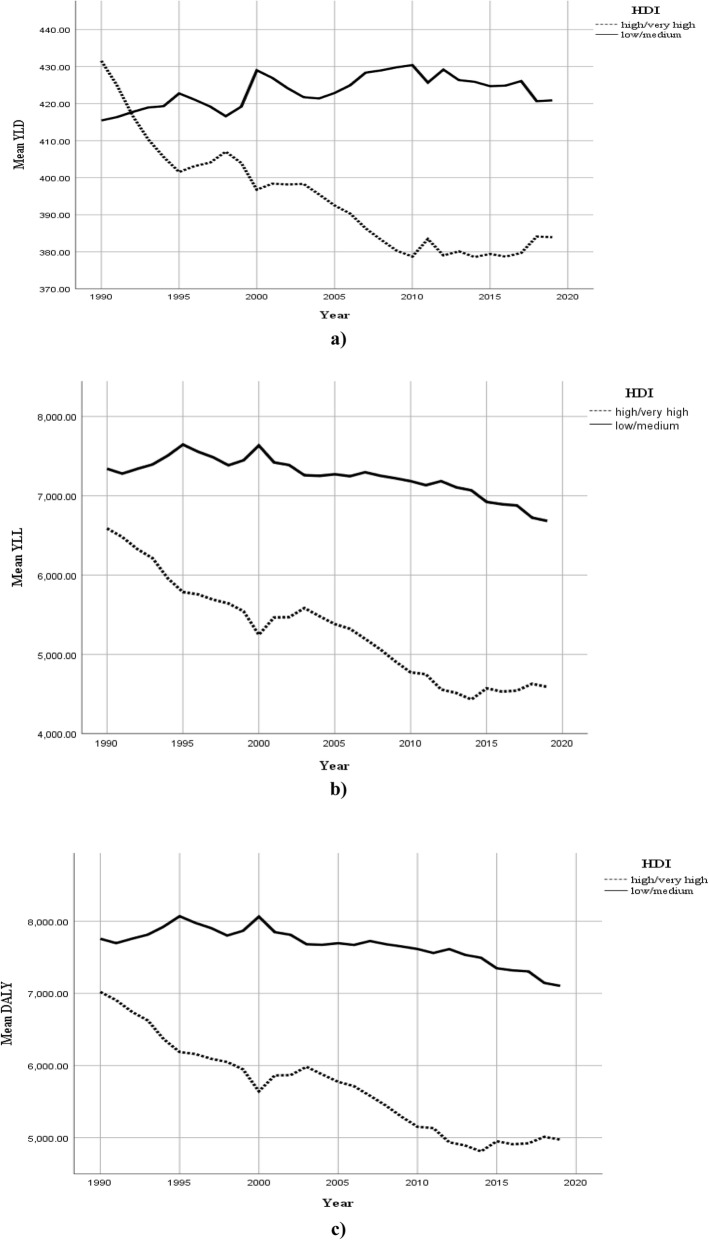
Global trend of CVD burden by HDI level from 1990 to 2019; **a**) YLD, **b**) YLL and **c**) DALY

Table 3 and Fig. 3 show the trend of YLD, YLL and DALY rates by HDI level (low/medium vs. high/very high) between 1990 and 2019. One more time, two conclusions can be made by a brief look at Fig. 3. First, countries in both categories (low/medium and high/very high HDI level) have experienced a decreasing trend of YLL, YLD and DALY over the study period, except a quite small increase in YLD rate for the countries with low/medium HDI level. Second, countries with high/very high HDI level had steeper downward trends of CVD burden than those with low/medium HDI level. More formally, simple calculations reveal that countries with low/medium HDI level had 1.0% increase in YLD rate, 8.0% decrease in YLL rate and 8.4% decrease in DALY rate over this 30-year study period. In the same period of time, countries with high/very high HDI level have experienced 8.9, 29.8 and 29.0% decrease, respectively in YLD, YLL and DALY rates.

**Table 3:** Descriptive statistics for CVD DALY, YLD and YLL rates per 100,000 by HDI level from 1990 to 2019

Index	HDI	1990	1995	2000	2005	2010	2015	2019
YLD	**Low/Medium**	415.49 (394.85, 436.1)	422.86 (402.68, 443.05)	428.87 (409.64, 448.10)	422.58 (402.65, 442.51)	430.02 (409.68, 450.35)	424.15 (402.61, 445.69)	420.42 (399.14, 441.70)
	**High/Very High**	430.61 (403.92, 457.3)	400.33 (374.48, 426.18)	395.52 (370.38, 420.66)	391.58 (368.73, 414.42)	377.78 (357.52, 398.04)	378.79 (359.40, 398.18)	383.30 (363.86, 402.73)
	**Total**	431.30 (416.35, 446.2)	424.72 (409.71, 439.74)	421.01 (405.91, 436.11)	413.28 (398.21, 428.35)	406.43 (391.49, 421.37)	401.17 (386.30, 416.05)	399.46 (384.76, 414.16)
YLL	**Low/Medium**	7312.43 (6770.87, 7853.98)	7596.13 (7009.53, 8182.74)	7587.15 (6986.54, 8187.75)	7239.29 (6603.38, 7875.20)	7153.14 (6508.86, 7797.43)	6896.15 (6262.52, 7529.79)	6661.18 (6008.37, 7313.99)
	**High/Very High**	6474.75 (5815.08, 7134.42)	5693.09 (4955.63, 6430.54)	5178.49 (4525.78, 5831.19)	5296.77 (4617.81, 5975.73)	4699.76 (4152.07, 5247.44)	4515.92 (3987.84, 5044.00)	4539.95 (4043.62, 5036.27)
	**Total**	7334.95 (6940.15, 7729.74)	7220.13 (6775.23, 7665.03)	6754.28 (6306.46, 7202.10)	6385.05 (5910.94, 6859.17)	5863.24 (5417.46, 6309.02)	5508.33 (5078.31, 5938.34)	5304.35 (4895.49, 5713.21)
DALY	**Low/Medium**	7727.92 (7177.09, 8278.75)	8019.00 (7422.55, 8615.44)	8016.02 (7404.49, 8627.55)	7661.87 (7015.18, 8308.55)	7583.16 (6928.20, 8238.12)	7320.30 (6674.21, 7966.39)	7081.60 (6416.34, 7746.86)
	**High/Very High**	6905.36 (6225.10, 7585.62)	6093.42 (5336.42, 6850.41)	5574.01 (4902.06, 6245.95)	5688.35 (4992.64, 6384.05)	5077.54 (4515.29, 5639.79)	4894.72 (4353.79, 5435.64)	4923.24 (4413.21, 5433.27)
	**Total**	7766.25 (7362.92, 8169.58)	7644.85 (7190.89, 8098.82)	7175.29 (6718.03, 7632.55)	6798.34 (6314.86, 7281.81)	6269.67 (5814.29, 6725.06)	5909.50 (5469.88, 6349.12)	5703.81 (5285.30, 6122.32)

Table 4 shows the obtained estimates from fitting joinpoint regression models to YLD, YLL and DALY rates stratified by HDI level. Regarding the estimates for the YLD data, one can observe that there are five joinpoints in 1995, 1998, 2001, 2004 and 2008 for countries with low/medium HDI level which leads to six different time intervals with different trends. The estimated APCs show annual percent changes of 0.3, − 0.4, 0.8, − 0.6, 0.6, − 0.2 and 0.1 in YLD rates for these countries, respectively in the time intervals of 1990–1995, 1995–1998, 1998–2001, 2001–2004, 2004–2008 and 2008–2019. For countries with high/very high HDI level, the fitted model to YLD rates resulted in three joinpoints and four time intervals. The estimated APCs are − 1.6, − 0.2, − 0.7 and 0.1, respectively in the periods 1990–1994, 1994–2003, 2003–2010 and 2010–2019. The estimated AAPCs may help us to make a straightforward inference about the CVD YLD trends in countries with low/medium and high/very high HDI level. Countries with higher HDI level had an average annual decrease of 0.4 per 100, 000 in YLD rates between 1990 and 2019, while those in poorer areas have experienced no significant change in the same period of time.

**Table 4:** Results of the Joinpoint regression models for trend analysis of CVD DALY, YLD and YLL rates in total population of the world countries by HDI level from 1990 to 2019

Index	Segments	HDI (Low/Medium)		HDI (High/Very High)	
		Time interval	APC (95% CI)	Time interval	APC (95% CI)
YLD	**Trend 1**	1990–1995	0.3^a^ (0.1, 0.6)	1990–1994	−1.6^a^(− 2.1, − 1.0)
	**Trend 2**	1995–1998	− 0.4 (− 1.3, 0.6)	1994–2003	− 0.2^a^ (− 0.4, − 0.0)
	**Trend 3**	1998–2001	0.8 (− 0.1, 1.8)	2003–2010	− 0.7^a^ (− 1.0, − 0.4)
	**Trend 4**	2001–2004	− 0.6 (− 1.6, 0.4)	2010–2019	0.1 (− 0.0, 0.3)
	**Trend 5**	2004–2008	0.6^a^(0.1, 1.1)	–	–
	**Trend 6**	2008–2019	−0.2^a^(− 0.3, − 0.1)	–	–
	**AAPC**	1990–2019	0.1 (− 0.1, 0.2)	1990–2019	−0.4^a^ (− 0.5, − 0.3)
YLL	**Trend 1**	1990–1995	0.7^a^ (0.2, 1.3)	1990–2000	−2.0^a^ (− 2.3, − 1.8)
	**Trend 2**	1995–2013	−0.3^a^ (− 0.4, − 0.2)	2000–2003	1.7 (− 1.5, 5.1)
	**Trend 3**	2013–2019	− 1.0^a^ (− 1.4, − 0.6)	2003–2013	− 2.2^a^ (− 2.5, − 1.9)
	**Trend 4**	–	–	2013–2019	0.5 (− 0.1, 1.0)
	**AAPC**	1990–2019	− 0.3^a^ (− 0.4, − 0.2)	1990–2019	− 1.2^a^ (− 1.5, − 0.8)
DALY	**Trend 1**	1990–1995	0.7^a^ (0.2, 1.3)	1990–2000	− 1.9^a^ (− 2.2, − 1.7)
	**Trend 2**	1995–2013	− 0.3^a^ (− 0.4, − 0.2)	2000–2003	1.6 (− 1.5, 4.9)
	**Trend 3**	2013–2019	−1.0^a^ (− 1.4, − 0.6)	2003–2013	−2.1^a^ (− 2.4, − 1.8)
	**Trend 4**	–	–	2013–2019	0.5 (− 0.1, 1.0)
	**AAPC**	1990–2019	− 0.3^a^ (− 0.4, − 0.1)	1990–2019	− 1.1^a^ (− 1.5, − 0.8)

^a^ Significant at alpha = 0.05

**Fig. 4:**
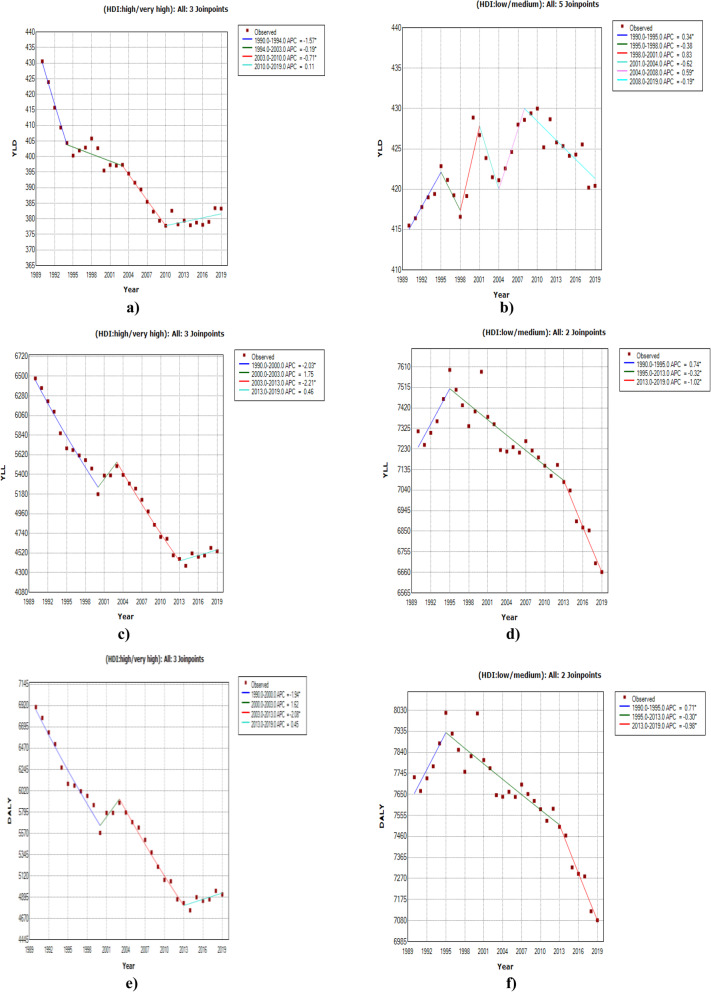
Results of joinpoint regression models for analyzing the global trend of CVD burden by HDI level from 1990 to 2019; **a)** YLD in high/very high HDI, **b)** YLD in low/medium HDI, **c)** YLL in high/very high HDI, **d)** YLL in low/medium HDI, **e)** DALY in high/very high HDI, **f)** DALY in low/medium HDI

Comparing the obtained results from fitting the joinpoint regression models for YLL and DALY rates in Table 4 indicates rather similar estimates. Therefore, we only interpret the estimates for the DALY rate data. In low/medium HDI countries, the fitted model resulted in two joinpoints and three different time periods. The significant estimated APCs are 0.7, − 0.3 and − 1.0, respectively in 1990–1995, 1995–2013 and 2013–2019. In high/very high HDI countries, three joinpoints with four time periods were found. The APC estimates are − 1.9, 1.6, − 2.1 and 0.5, respectively in the periods 1990–2000, 2000–2003, 2003–2013 and 2013–2019. Comparing the estimated AAPCs of − 0.3 and − 1.1 per 100, 000, indicate that the average annual percent reduction of DALY rate in wealthier regions was about 3.7 times of this reduction in poorer areas. Figure 4 displays the estimated trend of CVD YLD, YLL and DALY rates based on the fitted joinpoint regression models by HDI level over the period 1990–2019.

## Discussion

Nowadays, the pivotal role of cardiovascular diseases in diminishing the quality of life and increasing the global burden of disease is undeniable [7]. Despite our knowledge about significant impact of CVDs on global disability and mortality, limited studies have been previously conducted on analyzing the trend of CVD burden indices such as years of life lost, years of life with disabilities, and disability-adjusted life years. Also, the published studies in this field have not adequately focused on the role of important indicators of disease burden such as development and geographical location. In the present epidemiological study, we investigated the global trend of CVD YLL, YLD, and DALY in the last three decades. According to our findings, there is a worldwide downward trend in all three indices in this period of time. However, this reduction has occurred much faster in countries with higher HDI levels compared to those with low/medium HDI levels.

Based on the data from the GBD study 2019, the obtained results indicated that all the IHME super regions had fairly stable decreasing trend in YLD between 1990 and 2019. In addition, women had higher levels of YLD than men. Our literature review showed that there are few studies on assessing the long-term trend of CVD burden indices including YLL, YLD and DALY in the entire world. In a study by Roth et al., they reported a worldwide decreasing trend of YLD from 1990 to 2019 [3]. In another study, the researchers used a systematic analysis for the global burden of disease study 2019 and confirmed the global downward trend of YLD over the past three decades [7]. In addition, some researchers investigated the trend of CVD burden in different geographical locations over the past decades. For instance, Martinez et al. reported a descending trend of CVD YLD in the United States between 1990 and 2017. They also stated that rheumatic heart disease (RHD) had the sharpest downward slope among different types of cardiovascular diseases [11]. A worldwide study by Zimmerman et al. indicated that the global YLD of congenital heart disease (CHD) had a relatively stable pattern between 1990 and 2017. According to their findings, the Western and Central areas of the United States, Eastern sub-Saharan Africa, Central and Southeast Asia, China, and India have experienced the highest CVD YLD in the period 1990–2017. They also mentioned that among various types of cardiovascular disease, RHD and CHD were the leading causes for the increase of YLD [12]. A study in the Eastern Mediterranean counties revealed a fairly downward trend of CVD YLD in this region between 1990 and 2015.The obtained findings from this research shows that Oman and the United Arab Emirates had the highest and lowest levels of YLD at the beginning and ending year of the study, respectively [13]. Regarding the described researches in this field, it seems that global adoption of healthy lifestyles, controlling related risk factors (such as alcohol use, smoking, obesity, diabetes and hypertension), diagnosis of patient in the earlier stages and better access to effective treatments are the main reasons for decreasing trend of CVD YLD in the entire world [3, 7, 12–15].

Regarding our findings about CVD YLL trend, it is apparent that nearly all world countries have experienced a significant declining trend over the past three decades, with an annual mean reduction of more than 2000 per 100,000 individuals. Unlike the YLD, men had higher values of CVD YLL ​​than women in the period under the study. These findings are in agreement with some other published reports around the world [3, 12]. According to the obtained results from the study by Zimmerman et al., CHD YLL was more than twice of RHD YLL [12]. In the Eastern Mediterranean region, about 15% decrease in CVD YLL was reported in the period 1990–2015. Among the countries in this region, Afghanistan had the highest YLLs values, and Pakistan was the only country with an upward YLL trend in this period of time [13]. The reasons for observing this descending pattern in CVD YLL are quite similar to those mentioned for YLD trend [3, 15].

In this study, we also used the DALY index as a key measure for evaluating the burden of cardiovascular disease around the world. According to our findings, the global CVD DALY has continuously decreased over the study period. In addition, we found that men had higher DALY values than women, so that the difference between male and female populations was more than 1900 per 100,000 individuals at the ending year of the study. We also concluded that the DALY and YLL measures behaved similarly in the study period with a sharper decreasing slope compared to the YLD. Our findings are in line with the results of the study conducted by Roth et al. which reported a significant global downward trend of CVD DALY in the same time interval. According to their results, female population in Central Asia, Oceania, North Africa, Middle East, and Eastern Europe regions have experienced the highest CVD DALY in the past three decades. In addition, women in High-Income Asia Pacific, Australia and Western Europe had the lowest CVD DALY rates. They also demonstrated that male population in Central Asia, Eastern Europe and some parts of Oceania had the highest DALY rates and men in Australia, Western Europe, and Latin America have experienced the lowest CVD DALY rates between 1990 and 2019. According to their findings, Japan, France and Israel had the lowest CVD DALY rates, while parts of Oceania, Afghanistan and Uzbekistan had the highest DALY over the study period [3]. These results imply that the richer areas of the world are more likely to have lower CVD burden than those in poorer territories. It seems this regional diversity in the CVD burden reflects the differences in the awareness about this disease, controlling the potential risk factors, screening programs, availability of proper treatment, as well as the level of patients’ care in different populations [16, 17].

As a key objective of the present study, we assessed the relationship between HDI level and CVD burden indices. In this context, our statistical analysis revealed two main findings. First, there was a negative association between HDI level and CVD burden indices. In other words, we found that countries with higher HDI levels had lower rates of CVD burden indices than those with lower levels of HDI. Second, the results of joinpoint regression analysis showed that counties in both categories of HDI (Low/Medium and High/Very high) had a decreasing trend of CVD YLD, YLL and DALY in the past three decades, except for countries with Low/Medium HDI level which had an steady trend of YLD. However, areas with higher HDI have experienced remarkably steeper slope of downward trend than poorer regions over the study period. Although we did not find any published analytical article in the field of longitudinal relationship between HDI level and CVD YLD, YLL and DALY rates, there are a number of researches which give us some clues about this relation. For instance, in a cross sectional study based on the age-standardized mortality rates (ASMR) of non-communicable disease reported by the WHO in 2015, the researchers obtained an inverse correlation between HDI and ASMR. They concluded that countries with very high HDI had lower values of ASMR and premature mortality (before age 70 years) than countries with lower levels of HDI [18]. As discussed earlier, in a descriptive trend analysis study using the GBD 2019 dataset, Roth et al. showed that countries in wealthier regions of the world had lower CVD YLD, YLL and DALY rates compared to those with lower socio-economic status [3]. Finally, Amini et al. used the data from the GBD 2017 study to determine the longitudinal association between cardiovascular disease burden (including incidence and mortality rates) and HDI. They found that developed countries had sharper descending slope of incidence and mortality rates than developing countries [19]. In a study by Joseph et al., they declared that although the global CVD mortality has decreased in many parts of the world, the absolute number of deaths is growing in some other regions, especially in low and middle income countries [20]. Finally, Mensah et al. studied the potential causes of declining trend in cardiovascular mortality in the recent decades. They concluded that this declining trend is basically related to rapid progress in access to prevention, care and treatment as well as socio-economic status and geographic location [21]. In general, our findings and the reported results in all of the described studies show that the CVD burden is reversely associated with socio-economic status of the world countries.

Despite our above mentioned results about the negative relationship between CVD burden and HDI, it is important to pay attention to the findings from a different point of view. Regarding the estimated APCs in the last time intervals (segments) of the joinpoint regression analysis for both low/medium and high/very high regions (see Table 4), it can be found that the improvements in CVD burden metrics have started to stagnate (based on non-significant APCs) in countries with higher levels of HDI. Conversely, according to the estimated APCs for countries with lower levels of HDI, one can observe that these countries have experienced significant downward trends of CVD burden in the last time intervals. This pleasant finding for countries in regions with lower levels of development is in line with the reported results from a comprehensive analysis of GBD study 2019. In 2020, the GBD 2019 diseases and injuries collaborators presented a systematic analysis for the global burden of 369 diseases and injuries in more than 200 world countries and regions. They used the SDI as the measure of development and concluded that countries and territories with lower SDI levels have experienced steeper rate of change in age-standardized DALY rates for all causes (except HIV/AIDS, natural disasters, and war and conflict) over the last decade (in the period 2010–2019 compared with the two decades before) than those with higher values of SDI. They also stated that obesity, diminishing potential for more reductions in smoking, and lack of access to preventive and curative services by lower class of the societies are some of the most important reasons for recent stagnation or even upward trend in CVD burden in some regions with higher levels of SDI over the last decade [7]. On the other hand, more detailed comparison between the findings in the present research and the reported results in the above-mentioned manuscript reveals some differences. For instance, the reported global age-standardized CVD DALY rates in years 1990, 2010 and 2019 were, respectively, 7080, 5470 and 4860 per 100,000 in the described article. Although these numbers are fairly lower than our results, the computed percent change of − 31.4, i.e. $100\left(1-\frac{4860}{7080}\right),$ between 1990 and 2019 is relatively in agreement with our estimated AAPC of − 1.0 (Table 2) which results in percent change of − 30.0 for this thirty-year period. In general, a point by point comparison between our findings and the reported results in the described manuscript seems quite difficult, because of discrepancies in the statistical analysis approaches, stratification based on age groups and sex, methods of reporting the findings through relevant figures and tables and utilized time intervals for calculating different indices.

In the present study, we used powerful statistical methods for modeling the global trend of CVD burden by sex and HDI level. To our knowledge, this is the first work in this field which applies longitudinal statistical techniques for modeling long-term trend of CVD DALY and its components in nearly all world countries. This modeling approach (joinpoint regression analysis) allows us to divide the long-term period into smaller time intervals and estimate the pattern of change in outcomes under the study separately for each time interval. Utilizing the reported data from the Global Burden of Disease 2019 study as one of the most comprehensive and reliable data source in this field is another strength point of present study. At the same time, there numerous indicators which are statistically related with burden of CVD in different population. Ethnicity, smoking, alcohol consumption, obesity, high blood pressure and cholesterol, diabetes, dietary habits, inactivity, family history, indoor and outdoor air pollution, occupational exposures and socio-economic status are some of individual- and societal-level risk indicators of CVDs. Ignoring the concurrent effect of the described indicators on burden of CVD was the foremost limitation of our study.

In 2015, GBD introduced the SDI as a new metric to summarize the development status of world countries and territories. SDI ranges from 0 to 1 and can be computed as the geometric mean of total fertility rate under the age of 25, education for age 15 and older, and lag distributed income per capita [22]. Some researchers believe that this index makes inferences easier when the main objective of the study is to compare different health outcomes. However, in the current study, we preferred to use the HDI as the indicator of development since this index is more common than the SDI and most of the related articles in this field have generally utilized the HDI as a common metric for measuring development status of world countries. Moreover, in our latest paper about the relationship between development and trend of cardiovascular disease mortality, incidence, and mortality-to incidence ratio, we used the HDI as the development measure of world countries [19]. We also believe that these two indices (SDI and HDI) are highly correlated and fairly similar findings could be obtained by applying SDI as the summary metric of development.

## Conclusion

In general, our findings revealed that the CVD DALY and its components had a decreasing trend in the past three decades globally. This downward trend shows that the global efforts have been effective in controlling CVDs and their related consequences. Despite these hopeful results, comparing the slope of downward trend between countries with low/medium and high/very high HDI levels raises some concerns. It seems more developed countries have apparently experienced steeper declining slope of trend than poorer regions of the world. To reach the ambitious goals of the WHO’s 25 × 25 Global Action Plan both in developed and developing countries, there is an urgent need to develop relevant strategies for promoting the level of knowledge about this disease and its related factors, implementing efficient screening and preventive plans and facilitating the accessibility to proper care and treatment, especially in regions with lower levels of HDI.

## Data Availability

We confirm that all methods were performed in accordance with the relevant guidelines and regulations. The datasets analyzed during the current study are available in: GBD database: http://ghdx.healthdata.org/gbd-results-tool UNDP database: http://hdr.undp.org/en/content/database

## Abbreviations

CVD: Cardiovascular disease
NCD: Non-communicable disease
DALY: Disability-adjusted life years
YLL: Years of life lost
YLD: Years lived with disability
WHO: World health organization
SDI: Socio-demographic index
GBD: Global burden of disease
HDI: Human development index
UNDP: United Nations development programme
APC: Annual percent change
AAPC: Average annual percent change
RHD: Rheumatic heart disease
CHD: Congenital heart disease
ASMR: Age-standardized mortality rate
